# The clinical roles of generalists in Japan: A descriptive model of problem setting and problem‐solving

**DOI:** 10.1002/jgf2.70043

**Published:** 2025-06-30

**Authors:** Masayuki Amano, Takashi Watari, Taro Shimizu

**Affiliations:** ^1^ Department of Generalist Medicine Minaminara General Medical Center Oyodo Nara Japan; ^2^ Department of Diagnostic and Generalist Medicine Dokkyo Medical University Hospital Mibu Tochigi Japan; ^3^ Integrated Clinical Education Center Kyoto University Hospital Kyoto Japan; ^4^ General Medicine Center Shimane University Hospital Izumo Shimane Japan

“What do generalists do in clinical practice?” Students and specialists often ask this question. Although the identity of generalists in Japan is steadily forming, their specific roles in clinical practice remain unclear.

This ambiguity stems largely from the diverse nature of generalist practice in Japan. Generalists engage in a wide range of activities, including clinical practice, medical education, research, and management; in a range of settings including hospitals, clinics, and home care; in both urban and rural areas.^1^ Although this diversity is a strength, descriptions often emphasize differences rather than commonalities. Consequently, what generalists do in practice remains unclear.

This situation creates two important problems. For students, it can foster uncertainty about choosing a career as a generalist. For specialists, it can lead to confusion about when and how to collaborate with generalists, potentially hindering effective teamwork.

To address these challenges, we developed a model to clarify the common clinical roles of generalists (Figure 1). This editorial introduces the key aspects of the generalist's work in practice, focusing on the two fundamental processes of “problem setting” and “problem‐solving”.^2^

**FIGURE 1:**
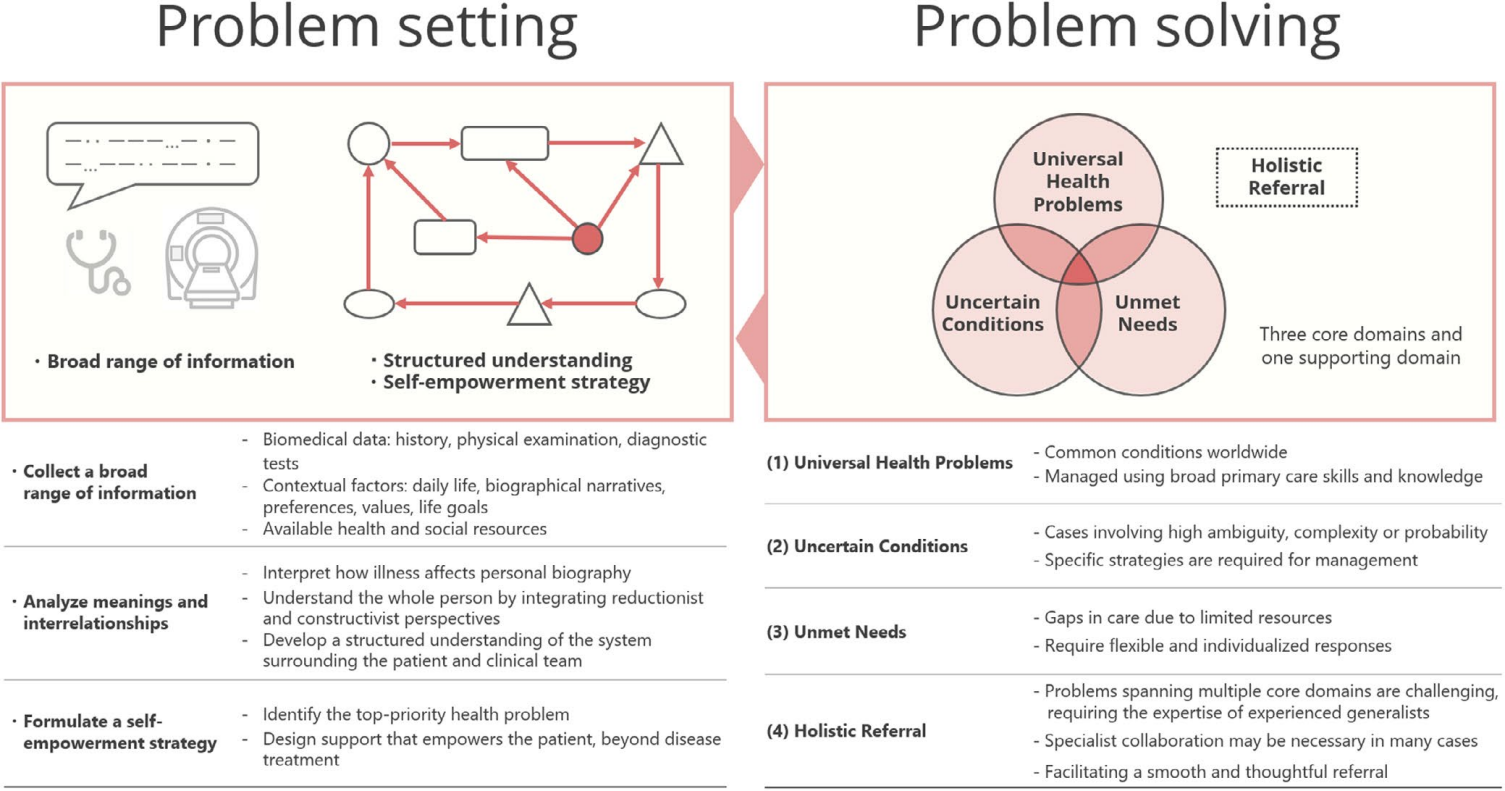
A descriptive model of generalist practice in Japan based on the dynamic interplay between problem setting and problem‐solving.

## 1 PROBLEM SETTING

Generalists fundamentally differ from specialists in their initial approach to patient care. Whereas specialists typically begin with a defined problem and apply evidence‐based strategies to engage in “problem‐solving,” generalists must first engage in “problem setting.” This is a complex process that involves identifying, prioritizing, and framing the patient's problems within their unique context before proceeding with problem‐solving. Although specialists may appear to engage in problem setting during diagnosis, their main role is to assess whether managing the problem falls within the scope of their specialty; thus, their first step is problem‐solving, not problem setting.

The process of problem setting starts with collecting a range of information extending beyond biomedical data from history taking, physical examination, and diagnostic tests, to include contextual factors such as daily life, the patient's health resources, biographical narratives, preferences, values, and life goals. The generalist then analyzes the interrelationships among these elements and works collaboratively with the patient to develop a shared understanding of the situation as a whole. Based on this holistic understanding, the healthcare team formulates a care strategy focusing on the most important health problem.

Problem‐setting skills have become a focus of academic inquiry within the fields of generalism and diagnostic excellence. “Patient‐centered medicine” stresses the importance of problem definition and proposes practical clinical methods.^3^ Furthermore, the “Craft of Generalism” highlights the role of interpretive dialogue and offers a framework for integrating different approaches such as reductionist and constructivist to develop a holistic understanding of the patient context.^4^ Drawing on these conceptual models supports the advancement of problem‐setting skills, highlighting the key role of structured, theory‐informed approaches in generalist practice.

## 2 PROBLEM‐SOLVING

To describe how generalists approach “problem‐solving,” we have organized their clinical activities into four domains (Figure 1):

1. *Universal health problems*: Generalists address common health problems such as hypertension, diabetes, aspiration pneumonia, and minor injuries, encountered in all regions and populations. Management is grounded in the application of primary care and primary healthcare knowledge across various disciplines. Responding to common problems is a defining area of generalist expertise.
2. *Uncertain conditions*: Generalists play a key role when the level of clinical uncertainty is high. This includes diagnostically challenging cases, multimorbidity, persistent physical symptoms, undifferentiated health problems, and situations requiring strong social support. Generalists adapt their strategies according to the level and drivers of uncertainty.^5^ Diagnostic reasoning and management of complex cases are core domains of generalist expertise.
3. *Unmet needs*: When medical needs arise in the community and no specialist is available, generalists provide crucial support, continuity, and compassionate care. Examples include home‐based care, working in rural areas, and the initial and stable‐phase management of specialized conditions in patients with limited access to specialist care. Generalists often provide comprehensive care across multiple fields, including new or rare conditions that fall outside traditional specialty boundaries, thereby addressing needs that might otherwise be fragmented by specialty focus. Responding to individual needs with flexibility is a hallmark of generalist expertise.
4. *Holistic referral*: Generalists provide care to all individuals but do not attempt to resolve every health issue independently. Even with universal health problems, specialist collaboration may be warranted depending on the severity. Facilitating appropriate referrals to ensure that patients receive optimal care in the right setting and coordinating integrated care are essential components of generalist expertise.

Of the four domains, the first three constitute the core areas of generalist practice. Although each domain requires specialized knowledge and methodologies, generalists develop these skills progressively through structured training. In clinical settings, many cases span multiple domains. As overlap increases, so does complexity, requiring the expertise of experienced generalists and highlighting the importance of continuous learning and professional development in generalist medicine.

## 3 A PRACTICAL ILLUSTRATION OF GENERALIST PRACTICE

Imagine a generalist seeing a middle‐aged woman presenting with elevated blood pressure (BP) in a clinic. The approach begins with problem setting. A comprehensive assessment reveals that the patient needs to be given a diagnostic workup for secondary hypertension. Through interpretive dialogue, the generalist understands the significance of the elevated BP within the patient's personal biography: she has lost a family member to a hypertension‐related disease and is therefore very anxious about her BP. Based on this structured understanding, if elevated BP is identified as a high‐priority health problem, the generalist then proceeds to the problem‐solving phase.

Initially, the elevated BP will be addressed as a universal health problem. If the cause is determined to be essential hypertension, lifestyle modification, support in coping with stress, and medication will be considered, if needed, within the scope of universal health problems. If the etiology remains unclear, the case may fall within the overlapping area of universal health problems and uncertain conditions. When factors such as multiple complex comorbidities and/or unfavorable social conditions such as financial hardship or rural living are present, the condition may span all three core domains. If the condition is difficult to manage, the patient may be referred to another generalist at a secondary or tertiary care center. If specialist care is required, such as for an underlying condition such as a pheochromocytoma, the patient is referred to the appropriate specialist, demonstrating holistic referral. In all these instances, the generalist's expertise is applied in a continuous manner.

## 4 CONCLUSION

“What do generalists in Japan do in clinical practice?” We have answered this question by proposing a model in which generalists apply their expertise to both problem setting and problem‐solving, continuously optimizing care through dynamic transitions between these two approaches.

This description of generalist work, by delineating the processes of problem setting, the four domains of problem‐solving, and their interaction, has several important implications. First, by providing a clearer understanding of the profession, it can reduce hesitation among students and young physicians considering a generalist career. Second, it can promote collaboration with specialists by clearly defining the scope and nature of generalist expertise, thereby facilitating more effective interdisciplinary teamwork. Finally, the versatility of this model extends beyond clinical care and has the potential to be applied in other areas, such as hospital administration and community health, facilitating the provision of more holistic care.

## AUTHOR CONTRIBUTIONS

Masayuki Amano led all aspects of the work, including conceptualization, methodology, investigation, writing – original draft, and project administration. Takashi Watari supported and supervised all aspects of the work, including providing guidance throughout the research and writing process. Taro Shimizu contributed to the conceptualization and supported the revision of the manuscript.

## CONFLICT OF INTEREST STATEMENT

Takashi Watari is an Editorial Board member of *Journal of General and Family Medicine* and a co‐author of this article. To minimize bias, they were excluded from all editorial decision making related to the acceptance of this article for publication.

## CONSENT FOR PUBLICATION

All authors have provided consent for publication.

## Data Availability

All relevant data are included in this report.
